# Nordic Walking training in BungyPump form improves cognitive functions and physical performance and induces changes in amino acids and kynurenine profiles in older adults

**DOI:** 10.3389/fendo.2023.1151184

**Published:** 2023-09-11

**Authors:** Ewa Rodziewicz-Flis, Ulana Juhas, Jakub Antoni Kortas, Joanna Jaworska, Ilona Bidzan-Bluma, Anna Babińska, Katarzyna Micielska, Małgorzata Żychowska, Giovanni Lombardi, Jędrzej Antosiewicz, Ewa Ziemann

**Affiliations:** ^1^ Department of Physiotherapy, Gdansk University of Physical Education and Sport, Gdansk, Poland; ^2^ Department of Bioenergetics and Physiology of Exercise, Medical University of Gdansk, Gdansk, Poland; ^3^ Department of Health and Natural Sciences, Gdansk University of Physical Education and Sport, Gdansk, Poland; ^4^ Department of Physiology, Medical University of Gdansk, Gdansk, Poland; ^5^ Department of Psychology, Gdansk University of Physical Education and Sport, Gdansk, Poland; ^6^ Institute of Psychology, Faculty of Social Sciences, University of Gdansk, Gdansk, Poland; ^7^ Department of Endocrinology and Internal Medicine, Medical University of Gdansk, Gdansk, Poland; ^8^ Department of Physical Education and Lifelong Sports, Poznan University of Physical Education, Poznan, Poland; ^9^ Department of Biological Foundations of Physical Culture, Kazimierz Wielki University in Bydgoszcz, Bydgoszcz, Poland; ^10^ Department of Athletics, Strength and Conditioning, Poznan University of Physical Education, Poznan, Poland; ^11^ Laboratory of Experimental Biochemistry and Molecular Biology, Istituto di Ricovero e Cura a Carattere Scientifico (IRCCS) Istituto Ortopedico Galeazzi, Milano, Italy

**Keywords:** exerkines, brain-derived neurotrophic factor, irisin, training physical, mental health

## Abstract

**Introduction:**

Although impacts of physical activity on cognitive functions have been intensively investigated, they are still far from being completely understood. The aim of this study was to evaluate the effect of 12 weeks of the Nordic Walking training with BungyPump resistance poles (NW-RSA) on the amino acid and kynurenine profiles as well as selected myokine/exerkine concentrations, which may modify the interface between physical and cognitive functions.

**Methods:**

A group of 32 older adults participated in the study. Before and after the intervention, body composition, cognitive functions, and physical performance were assessed. Blood samples were taken before and 1 h after the first and last sessions of the NW-RSA training, to determine circulating levels of exercise-induced proteins, i.e., brain-derived neurotrophic factor (BDNF), irisin, kynurenine (KYN), metabolites, and amino acids.

**Results:**

The NW-RSA training induced a significant improvement in cognitive functions and physical performance as well as a reduction in fat mass (p = 0.05). Changes were accompanied by a decline in resting serum BDNF (p = 0.02) and a slight reduction in irisin concentration (p = 0.08). Still, changes in irisin concentration immediately after the NW-RSA intervention depended on shifts in kynurenine—irisin dropped as kynurenine increased. The kynurenine-to-tryptophan and phenylalanine-to-tyrosine ratios decreased significantly, suggesting their possible involvement in the amelioration of cognitive functions. No changes of glucose homeostasis or lipid profile were found. Shifts in the concentrations of selected amino acids might have covered the increased energy demand in response to the NW-RSA training and contributed to an improvement of physical performance.

**Conclusion:**

Regular Nordic Walking training with additional resistance (BungyPump) improved cognitive functions and physical performance. These positive effects were associated with a reduced BDNF concentration and kynurenine-to-tryptophan ratio as well as changes in the amino acid profile.

## 1 Introduction

Nordic Walking (NW) training is a safe physical activity (PA), very popular among older adults. Its pro-health effects are well-documented, including improved cardiorespiratory fitness, physical performance (1), and cognitive functions (2). These beneficial changes are particularly evident among previously inactive elderly, especially in “The Covid-19 new Era” (3). In elderly, limited PA is often accompanied by a low-grade inflammation together with an impaired insulin sensitivity that altogether may intensify a decline in cognitive functions (4). Although the mechanisms induced by PA potentially acting on cognition have been intensively investigated, research gaps persist.

Available data indicate that an improvement in cognitive functions may be related to the circulating levels of myokines (5) and exerkines (6), released in response to skeletal muscle contractions. Among several myokines/exerkines, brain-derived neurotrophic factor (BDNF) and irisin are particularly relevant. BDNF regulates not only neurogenesis but also lipid and glucose metabolisms in both the central nervous system and the periphery (7, 8). Its concentration might be modulated by lactate or insulin-like growth factor 1 (IGF-1) (9). A study on animal models showed that irisin is a possible inducer of BDNF expression since it can cross the blood–brain barrier (10, 11). Results from human studies, however, did not show any consistent or direct relationship between BDNF and irisin. It is worth noting that in rats, approximately 70% of irisin is derived from muscle secretion whereas adipose tissue provides the remaining 30% (12), whereas in humans, the contribution of adipose tissue is much lower (13). Irisin not only is linked to the metabolic profile but also seems to be involved in the pathogenesis of conditions like sarcopenia, osteoporosis, and cardiovascular disease. In these conditions, low-grade inflammation (LGI), often encountered in the multimorbid elderly (14), is a common denominator.

The anti-inflammatory effects of exercise and potentially its beneficial effects of counteracting LGI are related to changes in myokine/exerkine concentrations as well as tryptophan metabolism. Tryptophan (Trp) may be converted into serotonin, a neurotransmitter involved in mood, anxiety, and cognition (15). However, Trp is mostly addressed to the kynurenine pathway (KP) (16), and an inflammatory status further stimulates this process. Kynurenine (KYN), upon accumulation in the central nervous system, increases neuroinflammation, which might lead to depressive traits and impair cognitive functions (17). Inflammatory cytokines, indeed, stimulate expression and activity of indoleamine 2,3-dioxygenase 1 (IDO-1), which catabolizes the conversion of L-tryptophan into KYN (18). The associations of KYN metabolites with cognition have been previously studied among people with cognitive or mental disorders. Increased levels of neurotoxic species 3-hydroxykynurenine (3-HK) and quinolinic acid (QUIN), along with a decrease in their neuroprotective counterpart, i.e., kynurenic acid (KYNA), may be involved in the pathogenesis of Alzheimer or Parkinson diseases (16). Thus, an imbalance in the KP is a significant contributor to neuroinflammation, which has significant meaning in human aging. Age is positively associated with KYN, QUIN, and kynurenine-to-tryptophan ratio (KYN/Trp). QUIN and KYN/Trp are also related to mortality and frailty among elders (19). An increase in plasma KYN and KYNA may be caused by a high metabolic demand due to a single bout of exercise (20). Another study investigated changes in KP metabolites after 10 weeks of multimodal training and reported only a tendency of 3-HK to decrease among older adults at risk of dementia (21).

Different types of PA, like the high-intensity interval training (HIIT), dance or Tai-Chi, are known to effectively ameliorate cognitive functions. NW is a more accessible and relatively affordable activity for older adults. Different protocols of NW training (22, 23), including a different number of sessions (either three or five per week) (24), have been previously investigated to verify its beneficial pro-health effects. Recently, to increase the intensity of NW training, poles with an integrated resistance shock absorber (NW-RSA) have been used in research (25, 26). This modified NW activity is commonly called BungyPump training. So far, only few studies described the physiological effects of BungyPump training, specifically its impact on cognitive functions. One revealed that 16 sessions of both traditional NW and NW-RSA training did not significantly affect cognitive or mental health outcomes in postmenopausal women but enhanced cardiopulmonary efficiency (25).

In this context, the aim of this study was to investigate the relationships between KP and cognitive and physical functions in response to NW-RSA training. We hypothesized that NW-RSA would improve cognitive functions and physical capacity and that this improvement would result from changes in the circulating levels of myokines/exerkines and amino acids (AAs) as well as kynurenine metabolites. Based on an animal study that showed an elevated cerebral blood volume in response to exercise (27), we also assumed that changes in cognition induced by the NW-RSA training would be related to insulin sensitivity and glucose homeostasis.

## 2 Methods

### 2.1 Subjects

**Figure 1:**
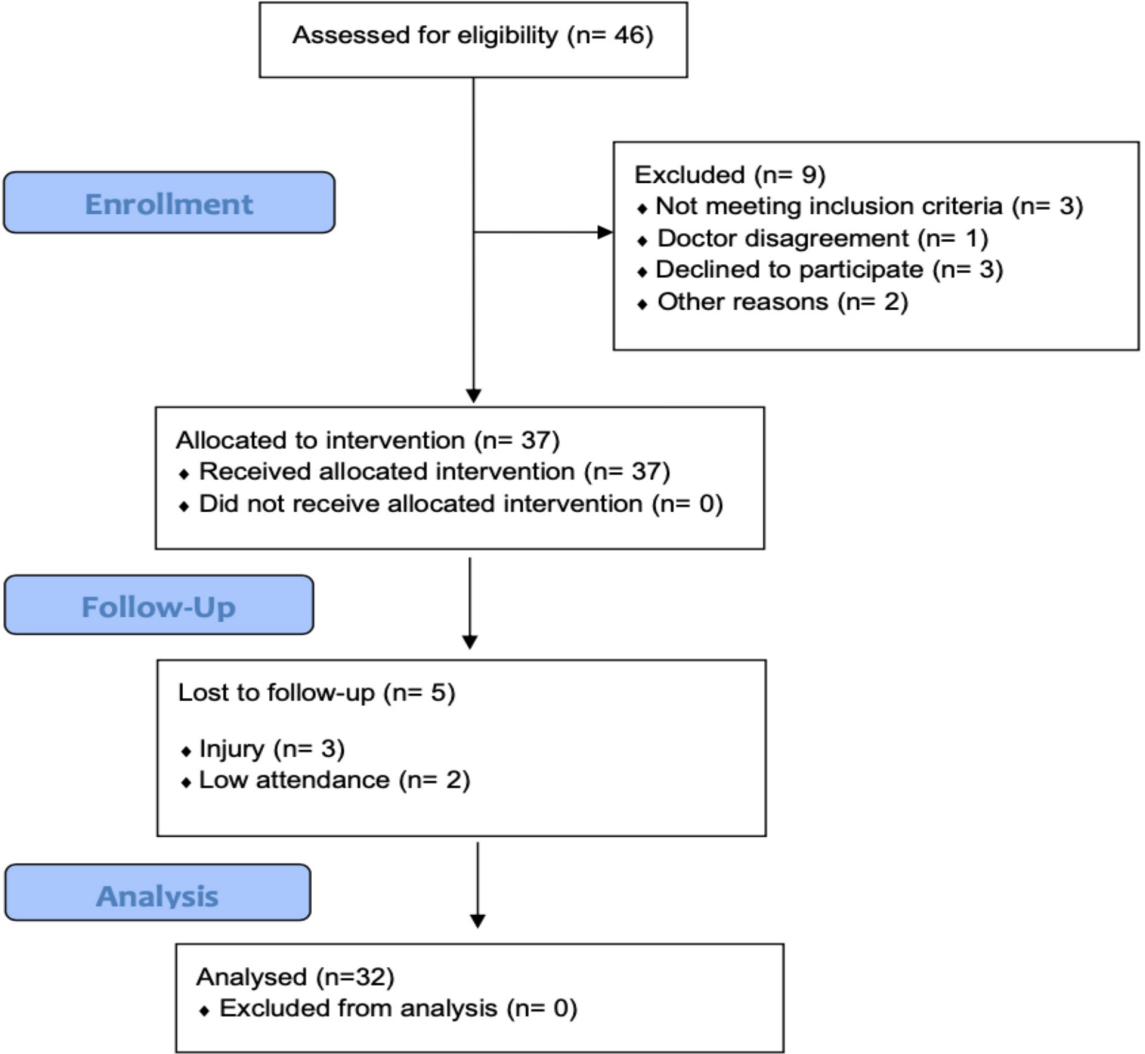
Flow diagram of the study.

Elderly subjects were recruited in local senior citizen clubs, third-age universities, and church communities. A group of 32 subjects (27 women and 5 men), aged 68.7 ± 6.0 years, took part in the experiment. Mean BMI (28 ± 4.9kg·m^−2^) indicated the overweight status of the study cohort. All participants were autonomous in their daily activities, without any severe cognitive or physical impairments. Volunteers were excluded if they were involved in any structured endurance exercise and/or had participated in any resistance exercise during the 6 months before the study. To exclude any form of dementia and cognitive impairments, the Mini-Mental State Examination (MMSE) was performed. Additional exclusion criteria were significant hip or knee problems, history of cardiac arrhythmia or unstable cardiovascular disease, neurological disease, dementia, neuromuscular disease, autoimmune disease, neoplasms, peptic ulcers, anemia, acute hernia, and diastolic blood pressure >100 mmHg. None of the participants were taking glucose-lowering and carbohydrate-stabilizing medications, and none had been diagnosed as diabetic. The cohort was asked not to change their diet and daily habits during the intervention period, as well as to refrain from introducing any nutritional supplementation. Recruitment details are presented in **Figure 1**. Participants were informed about the benefits and risks related to the protocol, and they provided their consent in writing. The study was conducted in accordance with the Declaration of Helsinki.

### 2.2 Study design

Participants performed NW-RSA training three times a week for 12 weeks. Two days before the first training session, the cohort was subjected to anthropometrical, psychological, and physical assessments. The same assessment was repeated at the end of the intervention. Neuropsychological tests were carried out to assess the cognitive functions such as attention, processing speed, verbal fluency, and executive functions, as detailed below. The physical performance tests determined physical capacity and functional mobility. In both instances (before and after the intervention), all participants were evaluated by the same coach and researcher-psychologist. Measurements were obtained in the morning between 9:00 and 10:00 am, after a light breakfast (the same for all participants).

In addition to the comprehensive assessment before and after the intervention, participants’ response to a single session of NW-RSA was monitored. Blood tests were done before and 1 h after the first and last session of NW-RSA.

### 2.3 Anthropometric measurements

Body mass and body fat were determined by dual-energy X-ray absorptiometry (DXA) performed on a Lunar Prodigy whole-body scanner (GE HealthCare, Madison, WI, USA) and enCORE v16 SP1 software (version 3.1.9.4, Heinrich Heine University, Düsseldorf, Germany). Assessments were performed early in the morning, after overnight fasting, before blood collection, usually within 1 h from the arrival for clinical assessment, and after medical check-up. Scanning mode was automatically chosen by the DXA apparatus. Participants were exposed to a radiation dose of approximately 2 μSv per scan; the scan took approximately 6–11 min. During DXA assessment, participants were lying on the scanning table in supine position, wearing light indoor clothing without any metal objects on their body (28). Additionally, the body mass index (BMI) was calculated.

### 2.4 Neuropsychological test battery

#### 2.4.1 Mini-Mental State Examination

All participants were carefully screened for cognitive impairment, to exclude any form of dementia, *via* the Mini-Mental State Examination (MMSE) (29, 30). The MMSE test also assessed cognitive functions in the areas of orientation, memory, attention and calculation, language, and visual construction. Higher MMSE scores correspond to better cognitive statuses and memory functions. The interpretation of results includes four categories: normal (25–30), mild dementia (20–24), moderate dementia (13–20), and severe dementia (<12). The overall Cronbach’s α for MMSE was above 0.80–0.95, demonstrating its high consistency (31).

#### 2.4.2 Geriatric Depression Scale-Short Form

The expression of any depressive symptoms was assessed using the Geriatric Depression Scale-Short Form (short form 15, GDS-15 item). The higher the score, the higher the level of depression is: no depression (0–4), mild depression (5–8), moderate depression (9–11), and severe depression (12–15) (32).

#### 2.4.3 Colour Trails Test

A Polish version of Colour Trails Test (CTT) was used to measure cognitive flexibility and processing speed (33, 34). The CTT is deemed a culture-free version of the Trail Making Test and consists of two parts: the first part CTT-I is used to assess the processing speed, visual-motor coordination, and attention. The second part CTT-II is used to assess additional executive functions. The examiner records the delay in completing each trial along with qualitative features of the performance indicative of brain dysfunctions, such as near-misses, prompts, number sequence errors, and color sequence errors (33, 34). The reliability of CTT is r = 0.64 for CTT-I and r = 0.79 for CTT-II. The overall Cronbach’s α demonstrated high consistency (33).

#### 2.4.4 Letter verbal fluency

Letter verbal fluency test was used to evaluate the cognitive status in aging. Participants had to name aloud as many words as possible beginning with the letter “K” (in Polish) in 60 s. Correct words were counted, excluding iterations and errors. Higher scores mean better functioning in processing speed and executive functions. Test–retest reliability was 0.70 (35).

### 2.5 Physical performance assessment

The 2000-m walking test was used to determine aerobic capacity (36). Before the test, verbal instructions were given to all participants. The test consisted of two stages: the reference phase, consisting of a 3-min warm-up (walk and stretching exercises), and the main test, consisting of 10 laps of 200 m each, performed on a flat surface. Time was measured using photoelectric cells (Racetime 2 SF, Microgate) with an accuracy of 0.001 s. The start was signalled by the coach.

The functional fitness was assessed using the Senior Fitness Test specifically designed for evaluating elderly. The test consists of six components: (1) 30-s chair stand, (2) arm curl, (3) chair sit-and-reach, (4) back scratch, (5) 8-foot up-and-go, and (6) 2-min step. The test follows a specified order, with a 1-min rest period between each component. Prior to the test, the cohort was familiarized with each component, except for the 2-min step (37).

### 2.6 Training program

The NW-RSA poles training program included 36 training sessions (three times per week, for 12 weeks). Each training session was performed in the following order: 10-min warm-up, 45–55-min NW-RSA training, and 10-min cool down (38). Special poles with an elastic resistance of 4 kg were used (Slimline Bungy Pump, Sports Progress International AB, Sweden). Each training session was supervised by a qualified NW instructor, who demonstrated and taught the proper walking technique with the RSA poles as well as monitored the intensity of the training workload. Each training was performed at 60%–70% maximal heart rate (HRmax), which was calculated during the supervised 2,000-m walking test (39). To monitor the intensity of the exercise, Garmin Forerunner 405 with a built-in GPS and an additional HR sensor was used. Only participants with at least 90% attendance, were considered to have completed the intervention.

### 2.7 Blood collection and analysis

Blood samples were collected before and 1 h after the first and last NW-RSA sessions, at 7:00–8:00 am, in fasting conditions. Blood was drawn from the antecubital vein into vacutainer tubes (Becton Dickinson, USA), by a professional nurse, and then centrifuged at 2,000*g* for 10 min at 4°C, to obtain serum that was stored at −80°C until assayed. An additional sample was collected to be analysed on BIOSYSTEMS apparatus (BIOSYSTEMS S.A., Spain) for hematological indicators.

Blood samples were assayed for kynurenine metabolites, amino acids, lipid profile, glucose concentration, and selected myokine/exerkines. The average intra-assay coefficient of variability was <10% for all measurements.

Serum concentrations of BDNF and irisin were assessed using sandwich ELISA assays, performed according to the manufacturer’s instructions (R&D Systems, USA; catalog no. DBD00, Phoenix Pharmaceutical Inc., USA; catalog no. EK 067-16, respectively). The precision performances, i.e., intra-assay and inter-assay CV, were 6.2% and 11.3%, respectively, for BDNF and <10% and <15%, respectively, for irisin.

Serum concentrations of amino acids were determined by applying ion-pair reversed-phase high-performance liquid chromatography using tandem mass spectrometry (IP-RP HPLC-MS/MS, TSQ Vantage Thermo Scientific) (40).

KYN metabolites KYN, KYNA, 3-hydroxykynurenine (3-HK), quinolinic acid (QA), xanthurenic acid (XANA), picolinic acid (PA), and 3-hydroxyanthranilic acid (3HAA) were analysed using LC-MS/MS (41), performed in the Masdiag Laboratory (Warsaw, Poland). The following ratios, reflecting the kynurenine profile status, were calculated: KYN/Trp (marking the rate-limiting step catalysed by IDO-1), KYNA/KYN and QUIN/KYN (reflecting KAT and KMO activities, respectively), KYNA/QUIN (reflecting the balance between the two branches), and PA/QUIN (reflecting ACMSD activity).

Vitamin D and related metabolites were assessed, and their concentrations were associated with the results obtained from cognitive functions and physical performance tests. This included 25-(OH)D_3_ and 25-(OH)D_2_ (25-hydroxyvitamin D), 24,25-dihydroxyvitamin D_3_ (24,25-(OH)_2_D_3_), and 3-epi-25-hydroxyvitamin D_3_ (3-epi-25-(OH)D_3_). Quantitative analysis of vitamin D metabolites was performed using LC-MS/MS (QTRAP^®^ 4500, Sciex, Framingham, coupled with ExionLC HPLC system) (42).

Serum lipoproteins and lipid profile [total cholesterol (TC), high-density lipoprotein (HDL), non-HDL cholesterol, low-density lipoprotein (LDL), and triglycerides (TG)] were determined by commercially available kits using enzymatic methods (Alpha Diagnostics, Warsaw, Poland).

Glucose levels were determined using Cobas 6000 analyser (Roche Diagnostics) according to the manufacturer’s instructions. Insulin levels were measured using the immunoassay kit from DiaMetra (catalog no. DKO076). The intra-assay CV was ≤5%, and the inter-assay CV was ≤10%.

### 2.8 Statistical analysis

Statistical analysis was performed using Statistica 13.1 software. Graphs were created in GraphPrism 7 software. All values are expressed as the mean ± standard deviation (SD). Shapiro–Wilk test was used to assess the homogeneity of dispersion from normal distribution. For homogenous results, a paired t-test analysis was performed to identify significantly different results. For heterogeneous results, Wilcoxon signed-rank test was used. The delta (Δ) was calculated as the difference between measurement after intervention and at the beginning of the project. Effect size (ES) and 1-β error probability were determined by G*Power software, using *post-hoc* power analysis. Additionally, the confidential interval (95% CI) of the mean value was calculated. The significance level was set at p < 0.05. The relationships between variables were evaluated using Pearson correlation for normally distributed results and the Spearman’s correlation coefficient for non-normally distributed results. The required sample size was calculated using the G*Power software for one group at two measurement points, considering the effectiveness of the training in terms of improving endurance measured by the 2,000-m walk test with the resulting indices: ES = 0.06, α = 0.05, β = 0.9. The analysis showed that 32 participants should be recruited for the study.

## 3 Results

### 3.1 General outcomes

Adherence to the NW-RSA training programme induced beneficial changes in the body composition. The total fat amount, expressed in grams and as percentage of total body mass, decreased from 29,267.3 ± 9,143.8 g to 28,465.1 ± 9,580.5 g and from 39.9 ± 7.7% to 39.1 ± 7.9%, respectively (p = 0.01). Furthermore, visceral fat expressed in kg and cm^3^ changed significantly (from 1.3 ± 0.9 kg to 1.2 ± 0.8 kg and from 1,410 ± 998.0 cm^3^ to 1,299.5 ± 9.3 cm^3^; p = 0.03). Although skeletal muscle mass increased slightly, the gain was not significant (from 20.6 ± 3.6 to 20.8 ± 3.5 kg). Hematological parameters, lipid profile, and glucose concentration were not affected in response to the intervention. These data are thus presented in **Supplementary Table 1**.

**Figure 2:**
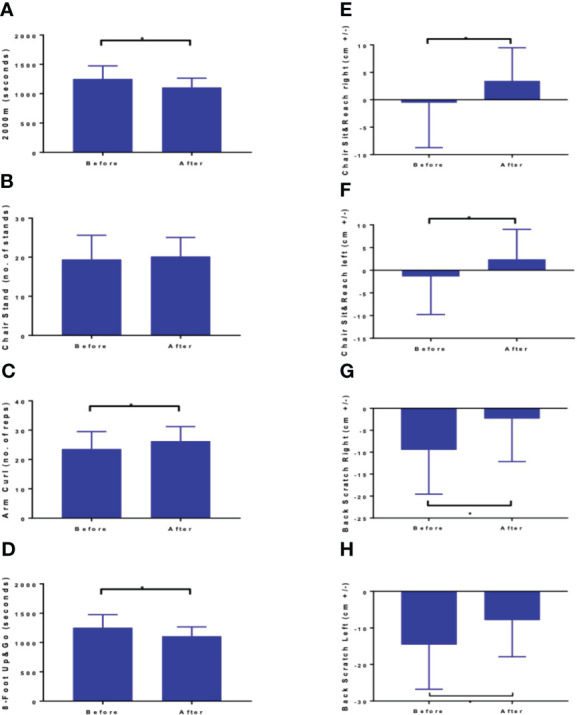
Results of fitness assessment: **(A)** 2000 m walk test **(B)** Chair Stand, **(C)** arm curl, **(D)** 8 foot up and go, **(E)** chair sit and reach right, **(F)** chair sit and reach left, **(G)** back scratch right, and **(H)** back scratch left z. Values are means ± SD. mean Δ – mean difference between measurement after and before the intervention; 95% CI -95% confidence interval of differences between two measurements difference between two measurements. Datastatistically significant at *p < 0.01 (except chair stand-no. of stands).

Completion of the NW-RSA training positively modified the physical performance. All values of Fullerton tests improved except for the number of chair stands remained unchanged (**Figure 2**). The concentration of 25-(OH)D_3_ did not change substantially—from 29.01 ± 12.5 ng·mL^−1^ at baseline, it stayed at 30.02 ± 9.9 ng·mL^−1^ after the intervention. Concentrations of vitamin D metabolites are presented in **Supplementary Table 2**.

### 3.2 Changes in selected amino acid concentrations and the kynurenine profile in response to the NW-RSA training

**Figure 3:**
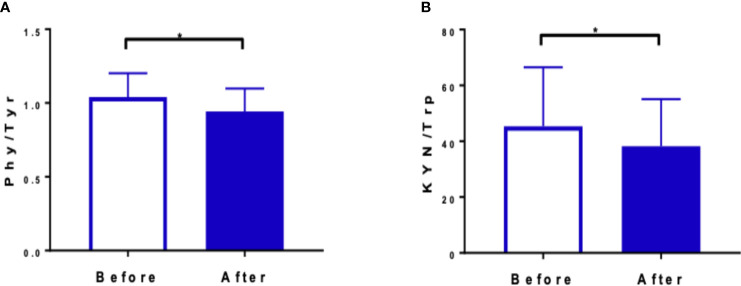
Ratios between phenylalanine to tyrosine **(A)** and kynurenine to tryptophan **(B)** before and after the training NW-RSA period. *Statistical significance between two time points.

**Figure 4:**
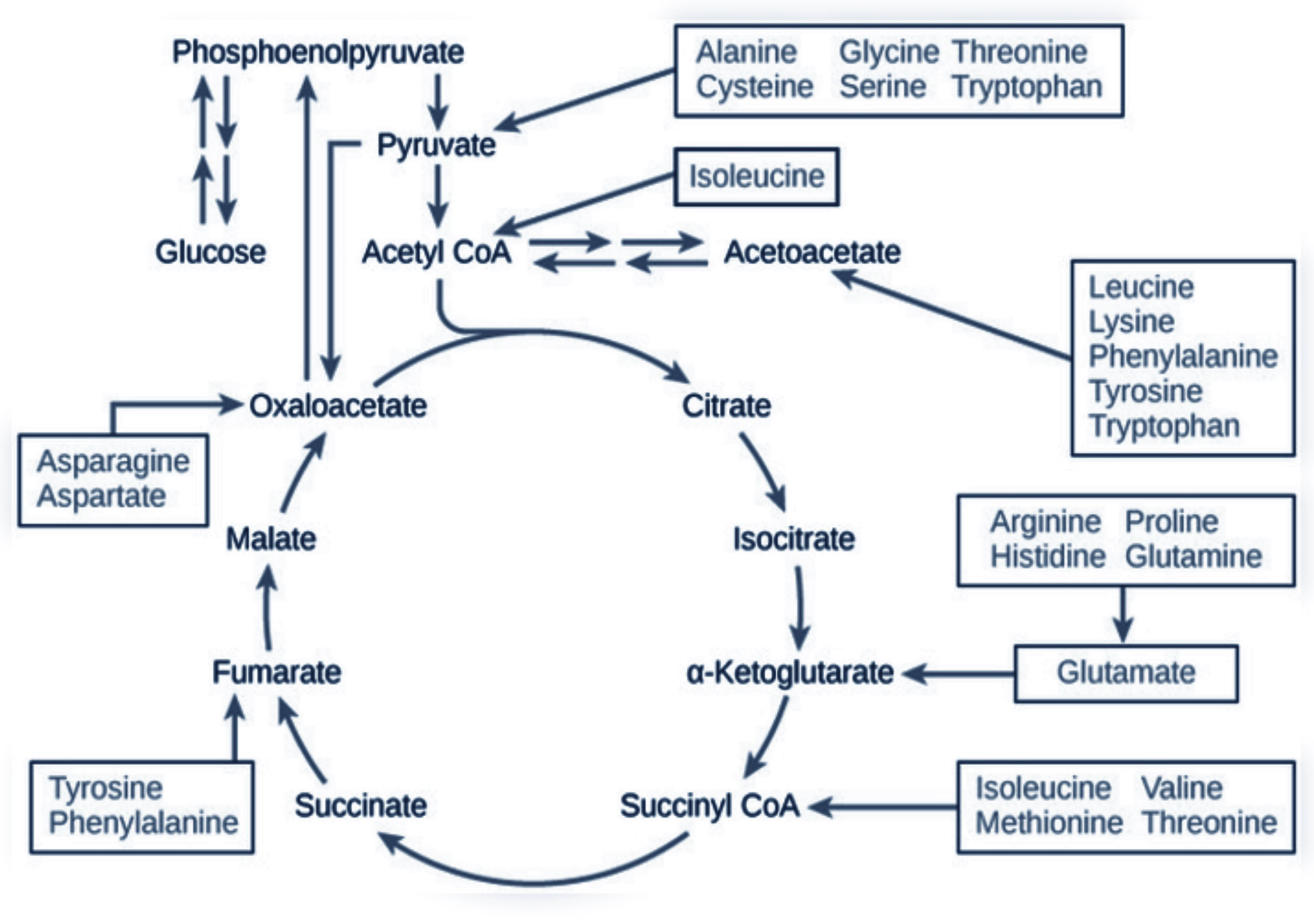
The role of amino acids in the Krebs cycle. Scheme made by the authors and created with BioRender.com. Down arrows indicate a decrease; up arrows indicate an increase in amino acid concentration; and horizontal arrow indicates unchanged concentration.

Changes in the amino acid profile are presented in **Table 1**. Amino acids were grouped by their role in metabolic pathways. Among the gluconeogenic precursors, a significant decrease was observed in alanine (p = 0.02). Glutamine (Gln) and glutamate (Glu) concentrations did not change significantly and the Gln/Glu ratio remained unchanged (4.46 ± 0.81 μM before vs. 4.40 ± 0.79 μM after the intervention). In the acetyl-CoA group of amino acids, a significant increase was recorded in lysine (p < 0.01), tryptophan (p < 0.01), and leucine (p < 0.01) concentrations. Isoleucine was the only branch amino acid, the concentration of which did not change. Changes in the concentrations of fumarate precursors included a significant decrease in asparagine (p = 0.01) and phenylalanine (p = 0.03) and an increase in valine (p = 0.03). No significant change was registered in tyrosine concentration, but the phenylalanine-to-tyrosine ratio (Phe/Tyr) was affected significantly (p < 0.01) (**Figure 3A**). In the α-ketoglutarate precursor group, a significant increase was observed in histidine, methionine, and sarcosine (p < 0.01, p = 0.01, p < 0.01, respectively). Proline and glycine concentrations remained unchanged. Among the pyruvate precursors, a significant decrease was observed only in serine (p < 0.01). Finally, among the remaining amino acid, a significant decrease in GABA concentration was observed (p = 0.03), even though it did not change after a single session of the NW-RSA training (**Supplementary Table 3**). Changes in amino acid concentrations in response to the NW-RSA training are shown in **Figure 4**.

**Table 1:** Changes in amino acid profile in response to NW-RSA training.

	Before	After	Δ (95% CI)	ES	1-β	p
Gluconeogenic precursors						
**Alanine [μM]**	422.29 ± 61.46	392.16 ± 57.81	-30.13 (-54.36; -5.9)	**0.45**	**0.69**	**0.02**
**Glutamate [μM]]**	130.30 ± 12.22	130.48 ± 18.79	0.19 (-5.37; 5.74)	0.01	0.05	0.95
**Glutaminate [μM]**	574.01 ± 74.46	564.23 ± 68.52	-9.78 (-29.67; 10.11)	0.18	0.16	0.32
Acetyl-CoA precursors						
**Lysine [μM]**	162.90 ± 15.43	181.93 ± 19.96	19.03 (11.43; 26.63)	**0.90**	**0.99**	**<0.01**
**Tryptophan [μM]**	48.50 ± 6.13	55.50 ± 4.8	6.99 (5.25; 8.74)	**1.44**	**0.99**	**<0.01**
**Leucine [μM]**	117.08 ± 16.42	126.03 ± 19.43	8.95 (3.32; 14.59)	**0.57**	**0.88**	**<0.01**
**Isoleucine [μM]**	62.31 ± 9.59	66.53 ± 10.97	4.22 (-0.01; 8.46)	0.35	0.50	0.05
Fumarate precursors						
**Asparagine [μM]**	21.32 ± 13.12	16.23 ± 12.59	-5.09 (-8.9; -1.27)	**0.48**	**0.75**	**0.01**
**Phenylalanine [μM]**	66.69 ± 9.28	63.02 ± 11.76	-3.68 (-6.89; -0.47)	**0.41**	**0.62**	**0.03**
**Valine [μM]**	246.65 ± 31.46	259.09 ± 34.09	12.44 (0.97; 23.91)	**0.39**	**0.57**	**0.03**
**Tyrosine [μM]**	64.92 ± 9.18	67.10 ± 10.06	2.19 (-2.37; 6.75)	0.17	0.16	0.34
α-Ketoglutarate precursors						
**Histidine [μM]**	75.55 ± 5.19	79.49 ± 5.56	3.94 (1.91; 5.97)	**0.70**	**0.97**	**<0.01**
**Methionine [μM]**	23.59 ± 2.79	25.90 ± 4.05	2.31 (0.59; 4.02)	**0.49**	**0.80**	**0.01**
**Proline [μM]**	178.21 ± 41.14	178.07 ± 39.92	-0.14 (-20.74; 20.45)	0.00	0.05	0.99
**Glycine [μM]**	295.78 ± 80.2	282.19 ± 70.4	-13.59 (-32.46; 5.28)	0.26	0.30	0.15
**Sarcosine [μM]**	2.32 ± 1.05	3.91 ± 2.05	1.59 (1.11; 2.08)	**1.18**	**0.99**	**<0.01**
Pyruvate precursors						
**Serine [μM]**	141.52 ± 22.32	123.64 ± 18.55	-17.88 (-26.51; -9.25)	**0.74**	**0.98**	**<0.01**
**Threonine [μM]**	111.56 ± 17.43	117.8 ± 13.27	6.24 (-0.37; 12.85)	0.34	0.46	0.06
AA engaged other pathways						
**GABA [μM]**	0.19 ± 0.06	0.17 ± 0.05	-0.02 (-0.04; 0)	**0.33**	**0.45**	**0.03**

Values are means ± SD. Δ, mean difference between measurement after and before the intervention; 95% CI, 95% confidence interval of differences between two measurements; ES, effect size; 1-β, power of the statistical test; GABA, γ-aminobutyric acid.

Bold values indicated statistical significance (p<0.05).

The response to the NW-RSA training did not affect kynurenine concentrations—the observed slight declines were not statistically significant. Also, KYN metabolites were not modified by the intervention. Still, the KYN-to-tryptophan (KYN/Trp) ratio dropped significantly (from 52.27 ± 12.33 nmol·L^−1^ to 44.06 ± 8.39 nmol·L^−1^; p < 0.01) (**Table 2**, **Figure 3B**). Changes in the kynurenine profile recorded 1 h after the first and last NW-RSA sessions are presented in **Supplementary Table 3**. Most of the changes were not significant. Only the concentration of quinolinic acid dropped significantly (p = 0.01).

**Table 2:** Changes in kynurenine metabolites post 12 weeks of NW training with shock-resistant absorbers poles.

	Before	After	Δ (95% CI)	ES	1-β	p
**3-Hydroxykynurenine [ng/mL]**	9.02 ± 1.15	8.91 ± 0.83	-0.11 (-0.46; 0.24)	0.11	0.09	0.52
**Kynurenine [ng/mL]**	521.26 ± 111.69	506.52 ± 91.73	-14.74 (-42.81; 13.32)	0.19	0.18	0.29
**Kynurenic acid [ng/mL]**	8.92 ± 3.13	8.47 ± 2.51	-0.45 (-1.17; 0.27)	0.23	0.24	0.21
**Quinolinic acid [ng/mL]**	98.06 ± 35.69	98.26 ± 34.36	0.20 (-9.51; 9.9)	0.01	0.05	0.97
**Xanthurenic acid [ng/mL]**	3.79 ± 1.44	4.02 ± 1.64	0.23 (-0.26; 0.71)	0.17	0.15	0.35
**Picolinic acid [ng/mL]**	6.22 ± 2.29	6.36 ± 1.89	0.13 (-0.47; 0.74)	0.08	0.07	0.66
**3-Hydroxyanthranilic acid [ng/mL]**	4.96 ± 2.93	4.53 ± 2.22	-0.42 (-1.3; 0.46)	0.17	0.16	0.34
**KYN/Trp [nmol/L]**	52.27 ± 12.33	44.06 ± 8.39	-8.21 (-11.48; -4.94)	**0.89**	**0.99**	**<0.01**
**KYNA/KYN [nmol/L]**	0.02 ± 0.01	0.02 ± 0.01	0 (0;0)	0.10	0.09	0.14
**QA/KYN [nmol/L]**	0.23 ± 0.05	0.24 ± 0.06	0.01 (-0.01; 0.02)	0.19	0.18	0.28
**KYNA/QA [nmol/L]**	0.09 ± 0.03	0.08 ± 0.03	0 (-0.01; 0)	0.28	0.33	0.13
**PA/QA [nmol/L]**	0.09 ± 0.04	0.10 ± 0.04	0 (-0.01; 0.01)	0.08	0.07	0.66
**KYNA/3HK [nmol/L]**	1.16 ± 0.32	1.12 ± 0.28	-0.04 (-0.13; 0.04)	0.18	0.16	0.33
**(XA+PA)/(KYN+3HK) [pmol/L]**	27.49 ± 8.17	29.43 ± 8.33	1.94 (-0.25; 4.12)	0.33	0.44	0.08

Values are means ± SD. Δ, mean difference between measurement after and before the intervention; 95% CI, 95% confidence interval of differences between two measurements; ES, effect size; 1-β, power of the statistical test; KYN, kynurenine, KYNA, kynurenic acid; Trp, tryptophan; QA, quinolinic acid; PA, picolinic acid; 3HK, 3-hydroxyanthranilic acid; XA, xanthurenic acid.

Bold values indicated statistical significance (p<0.05).

### 3.3 The impact of the NW-RSA training on selected myokine/exerkine concentrations

**Figure 5:**
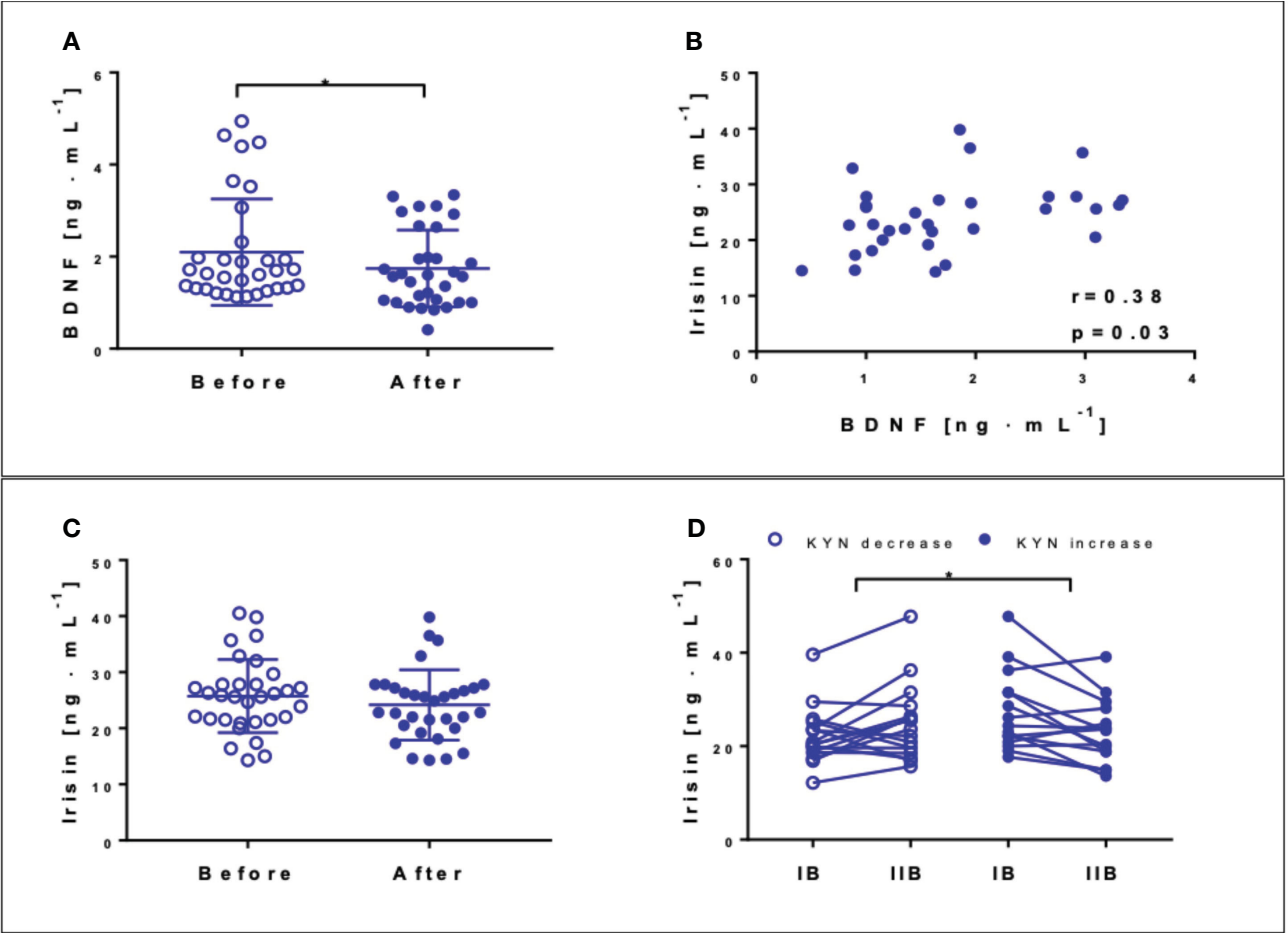
Concentrations of brain-derived neurotrophic factor (BDNF) **(A)** and irisin **(C)** at the baseline (blank circles) and after 12 weeks of NW-RSA (filled circles). Correlation between BDNF and irisin registered post-intervention **(B)**. Concentration of irisin 1 h after the first and last training sessions: IB at the baseline and IIB post-intervention—subjects exhibiting a drop (blank circles) and an increase in kynurenine in response to the whole intervention (filled circles) **(D)**. Data are presented as mean± SD. *Statistical significance in the group.

The resting BDNF concentration was reduced by 16.7% (p = 0.02) after the NW-RSA intervention (**Figure 5A**). The resting irisin concentration decreased slightly, from 25.7 ± 6.7 ng·mL−^1^ to 24.2 ± 0.08 ng·mL^−1^ (p = 0.08) (**Figure 5C**). The correlation between the resting irisin and BDNF concentrations recorded at the end of the intervention was significant (**Figure 5B**, p = 0.03). Interestingly, irisin concentration measured 1 h after the first and last sessions of NW-RSA differed vis-à-vis changes in kynurenine level. We observed an individual response to the intervention; in about half of the participants, kynurenine levels decreased after 12 weeks, whereas in the other tendency, it was the opposite. Among the participants that exhibited a decrease in kynurenine concentration, the concentration of irisin post exercise increased, whereas among those that exhibited an increase in kynurenine concentration in response to the intervention, the concentration of irisin decreased (**Figure 5D**).

### 3.4 The effect of the NW-RSA training on cognitive functions

Results from GDT revealed that none of the participants showed any depression symptoms. Average values registered at baseline were 3.13 ± 2.3 and remained unchanged at the end of the intervention 3.03 ± 2.3 (data not shown). MMSE scores indicated that the NW-RSA training induced a significant improvement in cognitive functions (p = 0.01) (**Table 3**). Beneficial changes were recorded in particular in short-term memory assessment (recall) (p = 0.04). Furthermore, CTT results at the end of the intervention indicated significant improvements in the processing speed, visual-motor coordination, and attention (p < 0.01).

**Table 3:** Changes in cognitive functions determined at baseline and after 12-week post of training intervention.

	Before	After	Δ (95% CI)	ES	1-β	p
**MMSE**	28.66 ± 1.23	29.16 ± 1.11	0.50 (0.16; 0.84)	**0.53**	**0.82**	**0.01**
**MMSE (results of short memory)**	2.38 ± 0.75	2.66 ± 0.55	0.28 (0.02; 0.54)	**0.38**	**0.56**	**0.04**
**TFS**	14.88 ± 5.63	15.25 ± 4.72	0.38 (-1.29; 2.04)	0.08	0.07	0.65
**CTT-I**	57.44 ± 25.12	46.09 ± 17.66	-11.34 (-18.38; -4.31)	**0.58**	**0.89**	**<0.01**
**CTT-II**	113.00 ± 38.69	104.72 ± 31.70	-8.28 (-18.72; 2.16)	0.29	0.35	0.12

Values are means ± SD. Δ, mean difference between measurement after and before the intervention; 95% CI, 95% confidence interval of differences between two measurements; ES, effect size; 1-β, power of the statistical test; MMSE, Mini Mental State Examination—results described in part I mean working memory, and results described in part II mean short memory; TFS, letter verbal fluency (TFS—abbreviation in polish language); CTTI, Color Trails Test—presented data in two parts described in section “Methods”.

Bold values indicated statistical significance (p<0.05).

## 4 Discussion

This study documents, for the first time, the impact of 12 weeks of the NW-RSA training, commonly known as BungyPump, on cognitive functions considering shifts in selected myokines/exerkines as well as the amino acid and kynurenine profiles. It finds that this special aerobic training programme ameliorated cognitive functions, namely, short memory, processing speed, visual-motor coordination, attention, verbal fluency, and executive functions. This overall improvement was associated with a drop in BDNF and resting irisin concentrations.

Previous studies reported a drop in BDNF in response to both a single session of and regular HICT (43) as well as after the NW training (25). Still, another study demonstrated opposite results after 11 weeks of traditional NW (1). BDNF has recently been discovered to serve as a soluble ligand of TrkB.T1 receptor, expressed by pancreatic β-cells, regulating insulin secretion in mammals (44). Another recent study demonstrated that folk dance training induced positive changes in the insulin resistance indicators in elderly subjects, accompanied by a drop in BDNF and an increase of irisin concentration (45). Therefore, the drop in BDNF concentration observed in our study may result from an enhanced uptake by the brain as well as its metabolic role. Still, contrary to the findings of Rodziewicz-Flis, we did not observe any changes in glucose homeostasis indexes even though the NW-RSA training significantly improved participants’ cognitive functions. Thus, our second hypothesis did not confirm. At the same time, changes in BDNF correlated with changes in irisin concentration.

Korkmaz and co-workers noted that irisin levels increased in response to 12 weeks of NW training but decreased in response to resistance training (46). Still, the authors did not assess the effects of the different training programmes on cognitive functions. Irisin changes in response to the NW-RSA training observed in this study may be related to changes in kynurenine–irisin concentration decreased in the participants, who experienced an increase in KYN. Although KYN metabolites were not significantly affected by the intervention, the resting KYN-to-Trp ratio declined at the end of the intervention, indicating that the indoleamine-pyrrole 2,3-dioxygenase 1 activity declined (47).

The role of irisin in cognitive functions in humans is still under exploration. Findings from 2019 described the role of FNDC5 in regulating synaptic function and memory in mouse models of Alzheimer’s disease (48) and of irisin in activating the canonical Notch signalling pathway to exert its neuroprotective effects (49). Irisin may bind to αV class integrin proteins and influence osteocyte functions in bone and adipocyte metabolism in adipose tissue (50). Among the participants of this study, a significant reduction in fat tissue occurred in response to the intervention. At the same time, a significant correlation was recorded between the concentrations of BDNF and irisin after the intervention, in line with the previously published data on animal models (10), where irisin induced the expression of BDNF in the hippocampus. Another study showed that circulating BDNF increased after a low-intensity cycling exercise, which was associated with an increase in circulating platelets (51). In this study, we observed a slight drop in platelets (**Supplementary Tables**), which may be linked with the drop in BDNF.

In both mice and humans, exercise upregulates PGC1α expression in skeletal muscle. It enhances the PGC1α-dependent muscular expression of kynurenine aminotransferase, an enzyme that converts neurotoxic KYN into neuroprotective kynurenic acid. In contrast to KYN, KYNA is not able to pass the blood–brain barrier. The imbalance between the neuroprotective KYNA and the neurotoxic KYN metabolites was proposed to be critical for the development of depression (52).

Together with the amelioration of cognitive functions, our study observed a decreased Phe/Tyr ratio. This signifies that the conversion of phenylalanine to tyrosine, which is catalysed by phenylalanine hydroxylase with participation of tetrahydrobiopterin (BH4), has been enhanced. A higher serum concentration of tyrosine (precursor of dopamine synthesis) was previously associated with better results in a cognitive assessment (53). In addition, the Phe/Tyr ratio was used as an indirect marker of BH4 status (54). Therefore, both the drop in Phe/Tyr ratio and the possible improvement in BH4 status in response to the intervention could be responsible for the amelioration in cognition. Importantly, the change in the ratios between the neuroprotective metabolites of KYN (xanthurenic acid and picolinic acid) and the neurotoxic metabolites (KYN + 3-hydroxyanthranilic acid), which can penetrate the blood–brain barrier, was close to being significant, confirming the potential neuroprotective effect of the intervention.

The shifts in some amino acid concentrations suggest that the energy demand increased in response to the NW-RSA training. Among other changes, the drop in alanine might suggest an increased uptake by the liver that ultimately might cover the energy demand and contribute to the improvement of physical performance. Also, the GABA (γ-aminobutyric acid) concentration declined, which might suggest its increased uptake in response to the intervention. It is the main inhibitory neurotransmitter, which plays a role in counterbalancing the action of glutamate, an excitatory neurotransmitter. Their balance is crucial in preventing anxiety and depression (55).

Furthermore, changes in the amino acid profile require further investigation. The data obtained in this study revealed that the concentrations of some AAs decreased whereas the concentrations of others increased. Serving as an energy source, AAs can be used for gluconeogenesis during catabolic states (56) and influence insulin and glucagon secretion (57). Increased levels of AAs have been observed in all stages of diabetes, including early prediabetic insulin resistance (58). A previous study indicated changes in AAs after military training, which combined endurance and resistance exercises, causing a decrease in tryptophan and an increase in arginine concentrations (59). On the contrary, in our study, even though tryptophan and BCAA concentrations increased, cognitive functions improved. Also, glutamine-to-glutamate ratio, potentially indicative of the overreaching state (59), remained unchanged, thus confirming that the applied workload was appropriate. This in turn may be associated with the observed enhancement of the physical performance. The diverging results may be due to diverse training modalities, intensities, and the participants’ age.

This study presents some limitations that further research should address. Only one group performed the NW-RSA training; however, recruiting participants for experiments became challenging in the post-pandemic period. Future studies should include a control group and follow-up to expand on the effectiveness of this training programme and determine how long beneficial changes sustain. Further research is also needed to determine the relationship between irisin and the kynurenine profile in response to exercise.

**Figure 6:**
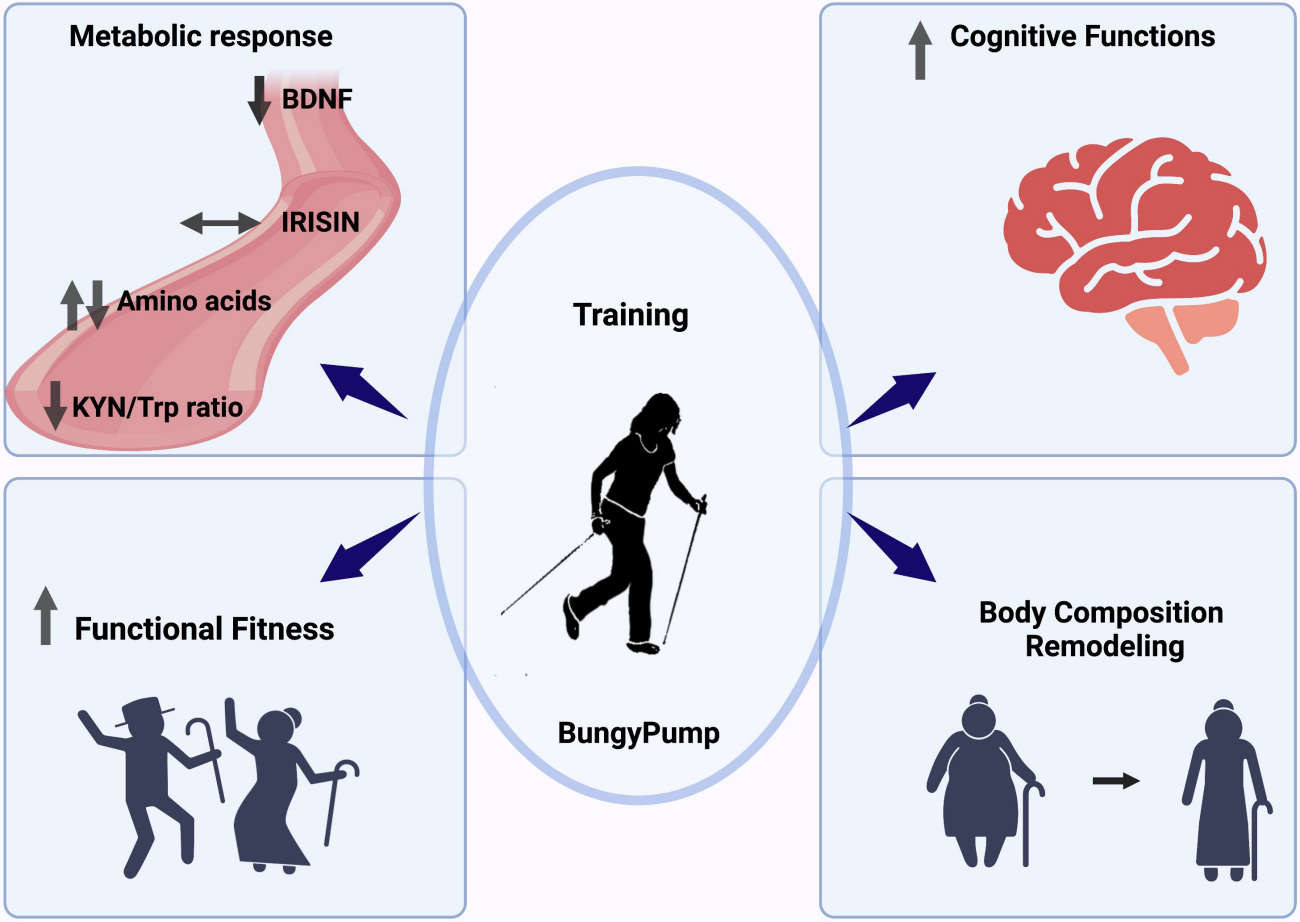
Graphical summary of the obtained outcomes.

In summary, regular BungyPump training led to the amelioration of cognitive and physical functions. These positive changes were likely mediated by reductions in BDNF concentration and KYN/Trp ratio (**Figure 6**).

## Data availability statement

The original contributions presented in the study are included in the article/**Supplementary Material**. Further inquiries can be directed to the corresponding author.

## Ethics statement

The studies involving humans were approved by Bioethical Committee of the Regional Medical Society in Gdansk (KB-34/18). The studies were conducted in accordance with the local legislation and institutional requirements. The participants provided their written informed consent to participate in this study.

## Author contributions

ER-F, UJ, GL, and EZ designed the study; ER-F, KM, JJ, AB, and MZ performed the research; ER-F, UJ, GL, JA, EZ, and JK wrote the paper; JJ, JK, and EZ analysed the data; and ER-F, JA, GL, JK, KM, JJ, MŻ, and EZ reviewed and edited the paper. IB-B analysed the data, reviewed, and edited the paper. All authors contributed to the article and approved the submitted version.
