# Supplementary material for: Nordic Walking training in BungyPump form improves cognitive functions and physical performance and induces changes in amino acids and kynurenine profiles in older adults
Source: Front Endocrinol (Lausanne). 2023 Sep 11;14:1151184. doi: 10.3389/fendo.2023.1151184 (PMC10520281; doi:10.3389/fendo.2023.1151184)
Supplement: Supplementary file 1 [file Table_1.docx]

Supplementary Table 1. Changes in hematological parameters in response to 12 weeks intervention of NW-RSA

|  | Before | After | Δ (95%CI) | ES | 1-β | p |
| --- | --- | --- | --- | --- | --- | --- |
| Thrombocyte [G/l] | 276.56±79.84 | 253.50±72.06 | -23.06 (-56.87; 10.74) | 0.25 | 0.27 | 0.17 |
| RDW-CV [%] | 13.48±0.81 | 13.23±0.73 | -0.26 (-0.43; -0.09) | **0.17** | **0.86** | **0.00** |
| MPV [fl] | 11.00±1.07 | 10.87±0.81 | -0.13 (-0.29; 0.04) | 0.28 | 0.34 | 0.13 |
| % neutrophils | 52.4±7.7 | 57..49±6.71 | 5..10 (3.09; 7.10) | **0.92** | **0.99** | **0.00** |
| % lymphocyte | 35.28±7.13 | 31.03±6.20 | -4.24 (-6.02; -2.46) | **0.86** | **0.99** | **0.00** |
| % monocyte | 9.18±1.76 | 8.32±1.39 | -0.87 (-1.37; -0.36) | **0.62** | **0.93** | **0.00** |
| % eosinophils | 2.40±0.96 | 2.51±1.33 | 0.11 (-0.2; 0.42) | 0.13 | 0.11 | 0.48 |
| % basophils | 0.76±0.34 | 0.72±0.29 | -0.03 (-0.12; 0.05) | 0.13 | 0.10 | 0.42 |
| neutrophils [G/l] | 3.47±1.80 | 3.48±0.92 | 0.01 (-0.71;0.73) | 0.01 | 0.05 | 0.99 |
| lymphocyte [G/l] | 2.11±0.79 | 1.85±0.5 | -0.27 (-0.41; -0.12) | **0.66** | **0.95** | **0.00** |
| monocyte [G/l] | 0.55±0.16 | 0.50±0.12 | -0.05 (-0.09; -0.02) | **0.56** | **0.86** | **0.00** |
| eosinophils [G/l] | 0.15±0.08 | 0.15±0.08 | 0 (-0.02; 0.01) | 0.01 | 0.05 | 0.65 |
| basophils [G/l] | 0.05±0.03 | 0.04±0.02 | 0 (-0.00; 1;0) | 0.01 | 0.05 | 0.14 |
| Lipid profile | | | | | | |
| Total cholesterol [mg/dl] | 210.81±55.81 | 197.13±43.99 | -13.69 (-28.66; 1.28) | 0.33 | 0.44 | 0.07 |
| HDL cholesterol [mg/dl] | 64±16.98 | 62.16±12.74 | -1.84 (-5.31; 1.62) | 0.19 | 0.18 | 0.29 |
| Non-HDL cholesterol [mg/dl] | 145.53±51.87 | 132.47±40.25 | -13.06 (-28.82; 2.7) | 0.30 | 0.37 | 0.10 |
| LDL cholesterol I [mg/dl] | 119.53±48.22 | 111.38±39.66 | -8.16 (-22.79; 6.48) | 0.20 | 0.20 | 0.26 |
| Triglicerides [mg/dl] | 126.34±72.42 | 110.22±32.21 | -16.13 (-41.11; 8.86) | 0.23 | 0.25 | 0.20 |
| Liver enzymes | | | | | | |
| ALT [U/l] | 27±22.09 | 22.91±13.98 | -4.09 (-8.56; 0.37) | 0.33 | 0.44 | 0.07 |
| AspAT [U/l] | 22.16±7.62 | 21.53±6 | -0.62 (-2.85; 1.60) | 0.10 | 0.09 | 0.57 |
| Glucose homeostasis indicators | | | | | | |
| Glucose [mg/dl] | 107.09±30.46 | 104.13±24.1 | -2.97 (-8.38; 2.44) | 0.20 | 0.19 | 0.27 |
| Insulin [µIU/ml] | 32.27±10.63 | 31.44±9.95 | -0.83 (-3.55; 1.88) | 0.11 | 0.09 | 0.54 |
| Values are means ± SD, Δ – mean difference between measurement after and before the intervention; 95% CI -95% confidence interval of differences between two measurements, ES – effect size, 1-β - power of the statistical test, RDW-CV - red blood cell distribution width – coefficient of variation, MPV - Mean Platelet Volume, HDL - High Density Lipoprotein, LDL - Low Density Lipoprotein, ALT - alanine aminotransferase, AspAT - aspartate aminotransferase. | | | | | | |

Supplementary Table 2. Vitamin D metabolites at baseline and after 12 weeks of NW-RSA training

|  | Before | After | Δ (95%CI) | ES | 1-β | p |
| --- | --- | --- | --- | --- | --- | --- |
| 25(OH)D3 [ng/mL] | 30.26±12.64 | 29.80±10.48 | -0.46 (-2.50; 1.58) | 0.08 | 0.07 | 0.65 |
| 25(OH)D2 [ng/mL] | 0.53±0.20 | 0.49±0.21 | -0.05 (-0.10; 0.01) | 0.33 | 0.45 | 0.09 |
| 24.25(OH)2D3 [ng/mL] | 2.96±1.88 | 3.00±1.70 | 0.05 (-0.21; 0.30) | 0.07 | 0.07 | 0.70 |
| 3-epi(OH)D3 [ng/mL] | 1.46±0.84 | 1.69±0.88 | 0.23 (0.06; 0.39) | **0.51** | **0.80** | **0.01** |
| Values are means ± SD. Δ – mean difference between measurement after and before the intervention, 95% CI -95% confidence interval of differences between two measurements, ES – effect size, 1-β - power of the statistical test. | | | | | | |

Supplementary Table 3. The comparison of values in selected proteins assessed 1h post first and last training session of NW-RSA

|  | I | II | Δ (95%CI) | ES | 1-β | p |
| --- | --- | --- | --- | --- | --- | --- |
| Exerkines | | | | | | |
| Irisin [ng/ml] | 24.58±7.64 | 24.01±7.5 | -0.57 (-3.08;1.93) | 0.08 | 0.07 | 0.64 |
| BDNF [ng/ml] | 21. 81±13.91 | 18.32±88. | -34.90 (-76.57;67.25) | 0.30 | 0.38 | 0.10 |
| Metabolites of kynurenine | | | | | | |
| 3-Hydroxykynurenine [ng/mL] | 9.67±1.06 | 9.76±0.85 | 0.1 (-0.18;0.37) | 0.13 | 0.11 | 0.49 |
| Kynurenine [ng/mL] | 517.11±131.85 | 542.93±94.48 | 25.82 (-11.56;63.19) | 0.25 | 0.28 | 0.17 |
| Kynurenic acid [ng/mL] | 10.49±4.01 | 9.76±2.65 | -0.73 (-1.81;0.34) | 0.24 | 0.27 | 0.18 |
| Quinolinic acid [ng/mL] | 107.1±32.94 | 98.96±24.32 | -8.13 (-14.34;-1.92) | **0.47** | **0.73** | **0.01** |
| Xanthurenic acid [ng/mL] | 5.4±1.5 | 5.56±1.28 | 0.16 (-0.3;0.61) | 0.13 | 0.11 | 0.49 |
| Picolinic acid [ng/mL] | 11.28±4.35 | 11.06±3.06 | -0.22 (-1.58;1.14) | 0.05 | 0.06 | 0.74 |
| 3- Hydroxyanthranilic acid [ng/mL] | 8.48±4.08 | 9.81±2.53 | 1.33 (-0.1;2.76) | 0.34 | 0.45 | 0.07 |
| Gluconeogenic precursors | | | | | | |
| Alanine [μM] | 406.74±78.88 | 412.98±56.95 | 9.45 (-13.13;32.03) | 0.15 | 0.13 | 0.40 |
| Glutamate [μM] | 138.65±26.34 | 141.19±25.43 | 1.57 (-7.86;11) | 0.06 | 0.06 | 0.74 |
| Glutaminate [μM] | 568.9±73.37 | 548.86±75.85 | -12.86 (-40.99;15.28) | 0.17 | 0.15 | 0.36 |
| Acetyl-CoA precursors | | | | | | |
| Lysine [μM] | 175.9±27.38 | 177.37±26.25 | 2.84 (-4.83;10.51) | 0.14 | 0.12 | 0.45 |
| Tryptophan [μM] | 58.47±10.95 | 62.95±8.61 | 4.5 (-0.46;9.47) | 0.33 | 0.44 | 0.07 |
| Leucine [μM] | 157.12±26.13 | 170.04±31.17 | 12.78 (3.71;21.85) | **0.52** | **0.81** | **0.01** |
| Isoleucine [μM] | 83.03±12.27 | 88.64±15.14 | 5.91 (0.11;11.72) | **0.37** | **0.53** | **0.05** |
| Fumarate precursors | | | | | | |
| Asparagine [μM] | 45.72±6.77 | 45.78±6.66 | 0.28 (-2.5;3.05) | 0.04 | 0.05 | 0.84 |
| Phenylalanine [μM] | 77.69±16.23 | 78.07±16.04 | -0.01 (-3.27;3.25) | 0.00 | 0.05 | 0.99 |
| Valine [μM] | 294.89±30.99 | 315.58±32.43 | 20.62 (11.8;29.45) | **0.86** | **0.99** | **0.00** |
| Tyrosine [μM] | 92.53±17.14 | 98.23±14.77 | 6.41 (1.33;11.49) | **0.46** | **0.72** | **0.02** |
| α-ketoglutarate precursors | | | | | | |
| Histidine [μM] | 82.52±9.04 | 83.96±6.89 | 1.52 (-1.87;4.9) | 0.16 | 0.15 | 0.37 |
| Methionine [μM] | 28.09±3.8 | 30.08±4.61 | 2.16 (0.31;4.02) | **0.43** | **0.65** | **0.02** |
| Proline [μM] | 249.13±61.26 | 265.86±47.32 | 20.49 (0.09;40.88) | **0.37** | **0.52** | **0.05** |
| Glycine [μM] | 251.7±58.46 | 243.31±51.33 | -2.51 (-15.78;10.75) | 0.07 | 0.07 | 0.70 |
| Sarcosine [μM] | 3.58±2.11 | 3.34±1.43 | -0.07 (-0.45;0.3) | 0.06 | 0.07 | 0.69 |
| Pyruvate precursors | | | | | | |
| Serine [μM] | 142.76±30.89 | 138.3±28.45 | -4.42 (-12.43;3.6) | 0.20 | 0.19 | 0.27 |
| Threonine [μM] | 115.09±16.8 | 123.70±20.23 | 9.67 (3.44;15.89) | **0.57** | **0.88** | **0.00** |
| AA engaged other pathways | | | | | | |
| GABA [μM] | 0.19±0.06 | 0.19±0.06 | 0 (-0.01;0.01) | 0.00 | 0.05 | 0.74 |
| Values are means ± SD. Δ – mean difference between measurement after and before the intervention; 95% CI -95% confidence interval of differences between two measurements, ES – effect size, 1-β - power of the statistical test, BDNF - brain-derived neurotrophic factor, GABA - gamma-aminobutyric acid | | | | | | |
